# Microbiota in Chronic Mucosal Inflammation

**DOI:** 10.4061/2010/395032

**Published:** 2011-01-11

**Authors:** G. Gerhard Rogler, Dirk Haller, Christian Jobin

**Affiliations:** ^1^Zürich, Switzerland; ^2^TU Munich, Germany; ^3^Departments of Medicine, Pharmacology, Immunology/Microbiology, University of North Carolina, 111 Mason Farm road, 7341B Medical Biomolecular Research Building CB No. 7032, Chapel Hill, NC 27599, USA

The human gastrointestinal tract is occupied by a complex and abundant microbial community reaching as high as 10^13^–10^14^ microorganisms in the colon. This microbiota participates in a symbiotic relationship with their eukaryotic host, and this partnership is viewed as essential for maintaining homeostasis. The microbiome contains a wealth of information, encompassing 150-fold as many genes as the human genome and performing essential and nonredundant tasks (e.g., nutrition/energy balance, immune system toning, and pathogen exclusion) for the host. Recent studies have hinted that a core human microbiome exists at the gene level, with a large number of microbial genes and pathways shared among individuals. Deviations from this core microbiome could potentially affect human health and promote disease state. The coexistence of the host with its intestinal microbiota is tightly controlled at various levels and an accumulating body of evidence suggests that the failure of this homeostasis is an important contribution to disease development. Recent discoveries clearly suggest that the gut microbiota of individuals throughout their lifespan is a powerful determinant of chronic diseases and that the mechanisms underlying this link involve the development of inflammatory activity. The microbiota has already been linked to cardiac development, angiogenesis, innate and adaptive immunity, metabolism, nutrient acquisition, and gastrointestinal development and homeostasis. Furthermore, alterations in microbial community composition are associated with multiple diseases, including obesity, fatty liver disease, type 1 and type 2 diabetes, kidney disease, arthritis, and inflammatory bowel diseases (IBD).

On the host side, a series of mechanisms help contain the formidable antigenic power of the microbiota. One of the main mechanisms, which could be regrouped under the wide umbrella of functional mucosal barrier, includes the formation of epithelial tight junction, mucus production, antimicrobial peptide secretion, and immunoglobulin A release. Equally important is the presence of a sophisticated repertoire of innate receptors, each recognizing specific conserved microbial patterns present on various microorganisms. Because the basic structure of bacteria is relatively conserved, eukaryotes have developed throughout evolution sensing systems to detect these bacterial signatures which include cell wall components, locomotion system, and nucleic acids. These microbial sensors are termed pattern recognition receptors (PRR-) and include retinoic acid inducible gene-I-like RNA helicases (RLH), C-type lectin receptors (CLR), nucleotide-binding domain leucine-rich repeat proteins (NLR), also known as Nod-like receptors, and Toll-like receptors (TLR). This arsenal of innate sensors plays a critical role in maintaining intestinal homeostasis through elimination of pathogenic microorganisms and preventing the formation of a dysbiotic microbiota. Interestingly, modulation of the gut microbiota and prevention of a dysbiotic state have become the focus of intense research. Indeed, various strategies including the introduction of prebiotic, probiotic, dietary compounds, and recently fecal transplantation to treat recurrent *C. difficile *infection have received special attention to treat various intestinal inflammatory disorders.

Clearly, one could appreciate the complexity of these host-microbe interaction and the deleterious consequences of an improper response to both the microbiota and infectious microorganisms. For example, Nod2 polymorph-isms causing a loss of bacterial recognition mechanisms have been identified as susceptibility factors for Crohn's disease. Moreover, NOD2 variants are also implicated in the development of gingivitis and Graft-versus-host disease after bone marrow transplantation. 

This special issue of the International Journal of Inflammation brings into focus the complexity of bacteria-host interactions and the impact of these interactions on intestinal homeostasis, and conversely in the development of various pathological conditions.

**Gerhard Rogler** **Gerhard Rogler** **Dirk Haller** **Dirk Haller** **Christian Jobin** **Christian Jobin**

